# Assessment of Preservice Music Teachers’ Multicultural Personality: Multicultural Music Education Perspective

**DOI:** 10.3389/fpsyg.2022.726209

**Published:** 2022-05-06

**Authors:** Shu Chen, Kwan Yie Wong

**Affiliations:** Department of Music, Faculty of Creative Arts, Universiti Malaya, Kuala Lumpur, Malaysia

**Keywords:** multicultural personality, music, education, preservice music teacher, assessment

## Abstract

This study examines preservice music teachers’ multicultural personality level and characteristics in the context of multicultural music education in China, focusing on the factors that influence the teachers’ multicultural personality traits. We surveyed 433 preservice music teachers who responded *via* the Multicultural Personality Questionnaire-Short Form survey instrument. The results indicated that the multicultural personality level of preservice music teachers was intermediate, mainly due to a deficiency in the Flexibility and Emotional Stability dimensions, which are necessary for dealing with cultural differences in the face of unknown multicultural conditions. In addition, we also found that the size of a teacher’s hometown and the years of studying and teaching are significant factors in shaping preservice music teachers’ multicultural personalities. Preservice music teachers whose hometowns are big cities have a higher multicultural personality level than those in rural areas. The more time a preservice music teacher spent studying and teaching, the higher the multicultural personality level. Gender, educational level, and experience playing musical instruments have no statistically significant effect on the multicultural personality of preservice music teachers.

## Introduction

Since the 20th century, the diversification of the world economy, politics, and culture has made music educators realize the importance of multicultural music education. Multicultural music education teaches students world music so that they can perform musical functions within the multicultural musical context of society (Wong et al., 2016). Understanding and respecting the world’s music culture also shapes students’ multicultural personality traits. Multicultural personality is a series of characteristics, attitudes, behaviors, and abilities that enable a person to adapt to the multicultural environment successfully. It affects individuals’ attitudes toward cross-culturalism and determines their ability to take constructive actions in a multicultural environment, and it is a personality ability that people must possess under globalization (van der Zee and van Oudenhoven, 2000, 2001; van der Zee et al., 2013). Developing future music teachers with multicultural personality traits and multicultural knowledge through multicultural music education has been a major focus of educators. With the context of globalization, it is an essential issue to train music teachers with multicultural personality traits.

China is a harmonious, stable multicultural mosaic, comprises a highly multilingual, religiously diverse, ethnically diverse, and culturally diverse (McCarthy, 2009; Leibold and Chen, 2014). China’s music education adheres to and pursues multicultural music education and training future talents with multicultural abilities for the development of multicultural personalities. To this end, the Chinese government has issued a series of education reform programs (The Ministry of Education of the People’s Republic of China [MOE], 2001, 2007, 2011; The Department of Sports et al., 2003). As a result, multicultural music education in China aims to cultivate students with knowledge and abilities in music, develop students’ cross-cultural competence, and shape outstanding multicultural personalities. Additionally, Chinese educators and practitioners have been working hard to explore and develop a multicultural music education model suitable for China’s multi-ethnic cultural Background (Wang, 2003; Guan, 2005; Ma, 2006, 2013; Qian et al., 2020; Wu, 2020; Wu and Luo, 2021). Compared with other multicultural countries, however, multicultural music education is still not fully implemented in China (Lasauskiene and Sun, 2019). This may be challenge to Chinese music teachers’ multicultural ability and wisdom, as they should exhibit multicultural personality traits to better influence and guide their students. Multicultural study and cross-cultural experiences develop intercultural competence and enhance multicultural personalities (Tracy-Ventura et al., 2016; Kim, 2017; Liang and Schartner, 2020). Therefore, multicultural education and training for preservice music teachers is needed in university education (Gunara and Sutanto, 2021). University education should provide preservice music teachers with opportunities to explore multiculturalism, shape multicultural personalities, cultivate cross-cultural competence, expand the scope of multicultural knowledge, and develop the willingness to apply multicultural understanding (Barnatt et al., 2019; Lukman et al., 2021; Plugina et al., 2021). Integrating multicultural education into the music teacher education system is significant to providing a broader perspective for developing school personality education and personal and professional values and attitudes (Lasauskiene, 2018).

Teachers are the designers, organizers, and implementers of teaching activities and essential influencers in the formation of students’ personalities. It is widely agreed that a teachers’ personality significantly influences their teaching behavior, teaching attitude, teaching achievement, academic achievement, and self-efficacy, and it also affects whether they can become successful and effective teachers (Eilam and Vidergor, 2011; Eryilmaz, 2014; Win and Yee, 2018; Shakeel et al., 2021). More importantly, teachers’ personalities have a profound influence on the formation of students’ personalities, academic performance, and future careers (Eilam and Vidergor, 2011; Peng, 2013; Eryilmaz, 2014; Khalilzadeh and Khodi, 2018; Lukman et al., 2021). Moreover, with globalization, teachers need to have multicultural personalities to effectively address cultural differences in their work, study, and personal lives. van der Zee and van Oudenhoven (2000, 2001) and van der Zee et al. (2013) designed the 40-item Multicultural Personality Questionnaire-Short Form (MPQ-SF), framing multicultural personality in five dimensions: (a) *Cultural Empathy* is the ability to empathize with the feelings, thoughts, and behaviors of individuals from a different cultural background; (b) *Open-mindedness* is an open and unprejudiced attitude toward different groups and toward different cultural norms and values; (c) *Emotional Stability* is the tendency to remain calm in stressful situations versus a tendency to show solid emotional reactions under stressful circumstances; (d) *Social Initiative* is a tendency to approach social situations actively and to take initiative; and (e) *Flexibility* is a tendency to regard new and unknown situations as a challenge and to adjust one’s behavior to the demands of new and unknown situations (van der Zee and van Oudenhoven, 2001). The five-factor structure supporting MPQ-SF through factor analysis research has been confirmed in different samples from many countries (Ponterotto, 2010). Among the teacher education projects that promote the multicultural competence of teachers, MPQ-SF is one of the research models used to test the results of multicultural education and measure the multicultural competence of preservice and in-service teachers (Sarraj et al., 2015).

Using different language versions of MPQ-SF, researchers generally believe that preservice teachers must have high-level multicultural personalities, which is the key to implementing multicultural education (Polat, 2009; Polat and Metin, 2012; Polat and Barka, 2014; Eskici, 2016). Polat (2009) found preservice teachers to have sufficiently multicultural personalities. However, they tend not to possess enough of the Emotional Stability dimension of multicultural personality, creating a tendency to change the level of multicultural education with the change of individual factors. Eskici (2016) indicated that preservice teachers have a high level of multicultural personality but are lacking in the dimension of Flexibility. However, studies conducted by Polat and Metin (2012) and Polat and Barka (2014) found most preservice teachers do not have a sound multicultural personality. Preservice teachers who have received teacher training programs and teachers with a higher level of multicultural personality possess a stronger ability to cope with cross-cultural conflicts (Polat and Metin, 2012; Polat and Barka, 2014). Multicultural personality plays a pivotal role in the process of multicultural education (Eskici, 2016). There is no doubt that music teachers must have comprehensive multicultural personalities to help students develop multicultural personality traits to better enable them to respect and appreciate the cultural differences of world music and realize multicultural music education.

As intrinsic factors, multicultural personality traits are affected by the teachers’ environment, life, study, work, and many other elements. For instance, Budaev (1999) found that women scored lower on Emotional Stability but higher on Cultural Empathy than men. Studies done by Eskici (2016) indicated that female prospective teachers have more Cultural Empathy and Emotional Stability than males. And Polat (2018) also indicated that male preservice teachers had higher mean values in the Open-mindedness and females had higher mean values in the Flexibility. In addition, 36 years and older prospective teachers have more Social Initiative, Open-mindedness, Flexibility than younger prospective teachers (Eskici, 2016). Polat (2009) found that no significant relationship between the preservice teachers’ size of hometown and their multicultural personality traits, and it is in line with the study of Polat (2018). Polat (2009) showed a statistically significant difference between teacher candidates’ undergraduate majors and Cultural Empathy, while Tortop (2014) found no statistically significant difference in the Cultural Empathy dimension for different undergraduate majors. Learning a musical instrument also has an important impact on personality, and students who learn instrumental music have stronger self-control and self-confidence (Kaplan, 1961; Sample and Hotchkiss, 1971). Those who learn different types of musical instruments have been shown to have various personality characteristics, such as emotional stability, anxiety, neuroticism, introversion, and openness (Mihajlovski, 2013; Vaag et al., 2018). However, some studies have shown that musical instrument learning experience and the efficacy of musical instrument performance have no significant effect on personality (Welborn, 2012; Mihajlovski, 2013; Girgi, 2017). Furthermore, job level, mother’s educational background, having social media account, and daily Internet use are essential factors for preservice teachers’ multicultural personalities (van der Zee and van Oudenhoven, 2000; Polat, 2018).

On the other hand, multicultural personality has an essential impact on individuals’ behavior, attitude, and cognition. For example, having a multicultural disposition positively correlates with psychosocial well-being (Brummett et al., 2007). Additionally, higher Cultural Empathy and Social Initiative may indicate higher emotional intelligence (Ponterotto et al., 2011). Multicultural personality influences college students’ attitudes toward different races (Korol et al., 2018), religious diversity (Gawali and Khattar, 2016), and international students (Ye, 2020). Multicultural personality traits help students to have intercultural abilities and multicultural effectiveness (Wu and Bodigerel-Koehler, 2013; Jičínská, 2014; Rosiers et al., 2014; Mikheeva, 2018; Olejárová et al., 2020). While the MPQ-SF measures multicultural personality traits, it also emphasizes essential multicultural skills that can help individuals play an influential role in a culturally diverse society (Sarraj et al., 2015). Obviously, there is still relatively little research on music teachers’ multicultural personalities, especially in China. Therefore, this study uses the MPQ-SF to investigate preservice music teachers’ multicultural personality levels in Sichuan Province, China and explore the effects of gender, size of hometown, educational level, years of studying and teaching, and experience playing musical instruments.

### Purpose of the Study

This study examined the multicultural personality level in terms of Cultural Empathy, Open-mindedness, Emotional Stability, Social Initiative, and Flexibility among preservice music teachers in multicultural music education. We also explored the relationship between preservice music teachers’ demographic characteristics (gender, size of hometown, educational level, years of studying and teaching, and experience playing musical instruments) and their multicultural personality traits. To achieve this goal, the following research questions were put forward:

1. What is the level of the multicultural personality traits Cultural Empathy, Open-mindedness, Emotional Stability, Social Initiative, and Flexibility among preservice music teachers in multicultural music education?
2. To what extent do the preservice music teachers’ gender, size of hometown, educational level, years of studying and teaching, and experience playing musical instruments (independent variables) correlate with the MPQ-SF dimensions (dependent variables)?

## Materials and Methods

### Participants and Procedures

The participants were 433 preservice music teachers from four Normal universities in Sichuan Province, China. The samples were 29.6% male and 70.4% female. Bachelor’s degrees were held by 86.6%, while 12.2% had master’s degrees, and 1.2% had a PhDs. Regarding the size of hometown, 11.5% were from metropolitan areas, 56.2% from urban areas, and 32.3% from rural areas. There were 75.5% with 1–5 years studying and teaching, 15.5% with 6–10 years, 3.5% with 11–15 years, 4.8% with 16–20 years, and 0.7% with more than 20 years. For the experience with playing musical instruments, 18.2% could play Chinese musical instruments, 36.3% could play Western musical instruments, 39.0% could play both Chinese and Western musical instruments, and 6.5% could not play any musical instruments.

The electronic questionnaires are created using the Questionnaire Star software and distributed to the sample *via* a link. Potential samples can browse them and choose to answer the survey. Participants had the right to be informed before answering and were given a randomly drawn thank you bonus upon completion. Permission was obtained from four university music schools for this study. The participant-reported electronic questionnaire took approximately 20 min or less and measured multicultural personality, as well as demographic information. IP addresses and the information provided by respondents in the questionnaire will also remain anonymous and confidential.

### Measure

This study used the MPQ-SF developed by van der Zee et al. (2013). The MPQ-SF is composed of 40 items, which are divided into five dimensions: (a) Cultural Empathy; (b) Open-mindedness; (c) Emotional Stability; (d) Social Initiative; and (e) Flexibility. Each dimension has eight questions. The typical question such as “Pay attention to emotions of others,” “Tries out various approaches.” Cronbach’s alpha coefficient of reliability for the scale was 0.77.

Subjects answered the questions on a five-point Likert-type scale, with the responses ranked as 1: Totally Not Applicable, 2: Hardly Applicable, 3: Moderately Applicable, 4: Largely Applicable, and 5: Completely Applicable. A Chinese bilingual translator translated the questionnaire and a native English speaker proficient in Chinese followed backtranslation procedure (Brislin, 1980), and the Chinese translation was then improved through discussion. Demographic information was also requested from preservice music teachers, such as gender, size of hometown, educational level, years of studying and teaching, and experience playing musical instruments.

### Data Analyses

In this study, quantitative methods were used to collate and analyze the collected data. The data were evaluated using the Statistical Package for the Social Science (SPSS) version 23.0, which included means, standard deviations, Pearson product-moment correlations, analysis of variance (ANOVA), and regression analysis to evaluate and analyze the collated data.

The mean and standard deviations of each dimension score and composite score were calculated to examine the preservice music teachers’ multicultural personalities. The Pearson product-moment correlation was calculated to explore the relationship between continuous variables (years of studying and teaching) and the MPQ-SF. The ANOVA was used to compare the categorical variables (gender, size of hometown, educational level, and experience playing musical instruments) with the MPQ-SF. Regression analysis was used to explore the *p*-value of the demographic characteristics on the MPQ-SF. A probability level of *p* < 0.01 and *p* < 0.05 were set for all tests of statistical significance. Cronbach’s alpha ranges from 0.789 to 0.948 for the five dimensions of the MPQ-SF.

## Results

### Preservice Music Teachers’ Multicultural Personality Traits Level

Descriptive statistics were computed for the five dimensions of the MPQ-SF (see Table 1). Scale scores were calculated by taking the scale mean, ranging from 1 to 5. The higher the score, the better the multicultural personality evaluation. The results revealed that the overall mean of the MPQ-SF scale reported by respondents was *M* = 3.36. Among the five dimensions, the mean score of Cultural Empathy was the highest (*M* = 4.20). The lowest mean score was for Flexibility (*M* = 2.21). The five dimensions of the MPQ-SF are ranked from highest to lowest as follows: Cultural Empathy (4.20), Open-mindedness (4.03), Social Initiatives (3.45), Emotional Stability (2.93), and Flexibility (2.21). The standard deviation shows a low degree of variability, with the five dimensions all less than 1.00.

**TABLE 1:** Means and standard deviations for multicultural personality questionnaire-short form.

Subscale	Mean	Standard Deviation
Cultural Empathy	4.20	0.60
Open-mindedness	4.04	0.67
Emotional Stability	2.93	0.62
Social Initiative	3.45	0.49
Flexibility	2.21	0.60
Composite Score	3.36	0.30

**FIGURE 1:**
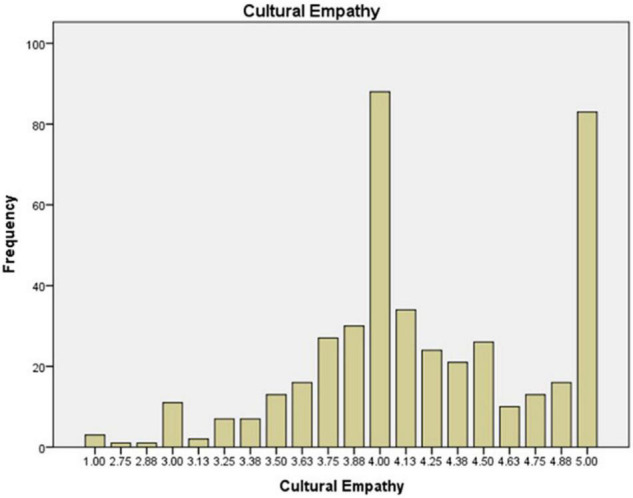
Cultural Empathy mean scores.

**FIGURE 2:**
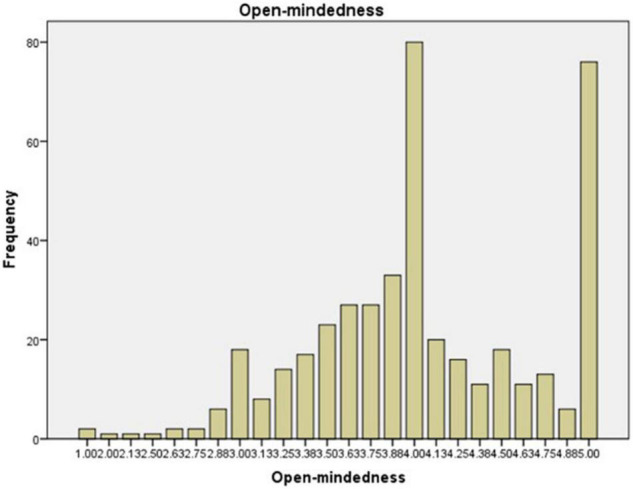
Open-mindedness mean scores.

**FIGURE 3:**
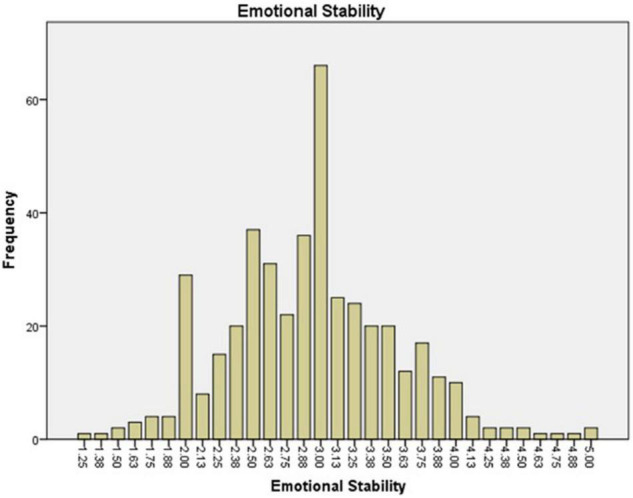
Emotional Stability mean scores.

**FIGURE 4:**
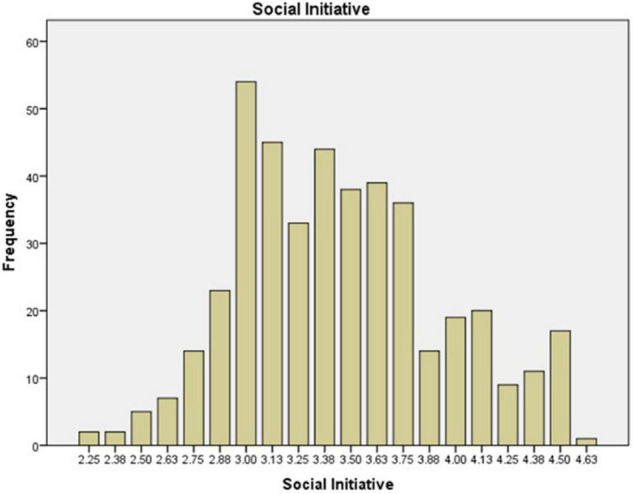
Social Initiative mean scores.

**FIGURE 5:**
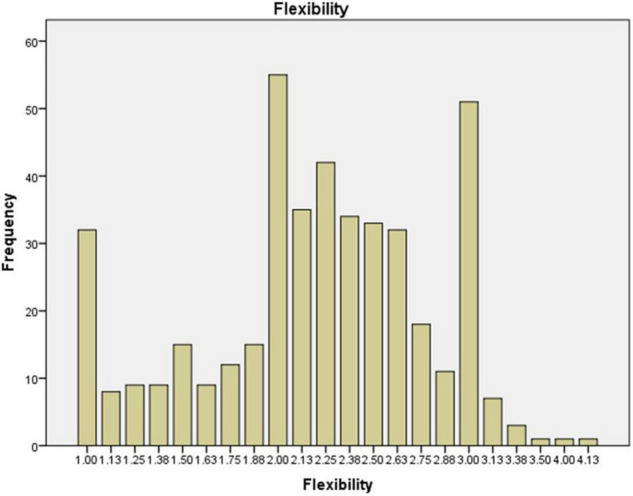
Flexibility mean scores.

**FIGURE 6:**
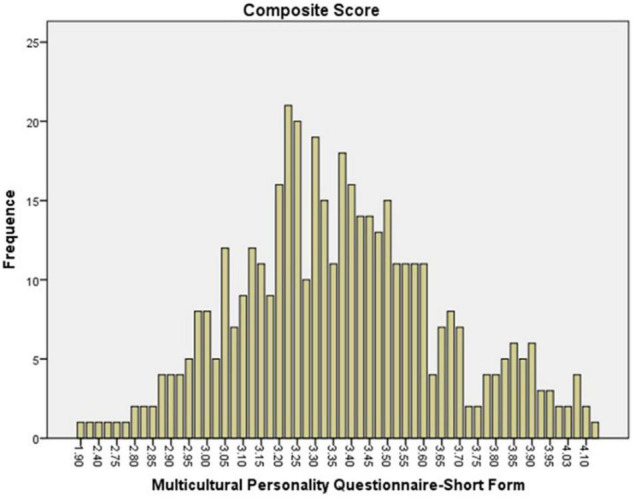
MPQ-SF mean scores.

The preservice music teachers’ mean value frequency for each scale is shown in Figures 1–6. Based on the mean score level: 1–2.32 “Low”; 2.33–3.66 “Mid-Range”; 3.67–5.0 “High” (van der Zee et al., 2013), the mean scores are interpreted as: Cultural Empathy was High, Open-mindedness was High, Social Initiative was Mid-Range, Emotional Stability was Mid-Range, and Flexibility was Low. The MPQ-SF composite scale is Mid-Range level. Therefore, in this study, the ranking of the multicultural personality traits reported by preservice music teachers is as follows: Cultural Empathy, Open-mindedness, Social Initiatives, Emotional Stability, and Flexibility.

### Relationship Between Preservice Music Teachers’ Demographic Characteristics With Multicultural Personality Traits

Table 2 shows how the preservice music teachers’ demographic characteristics correlate with the MPQ-SF. There was statistical significance between continuous demographic variables (years of studying and teaching) and the Open-mindedness (*r* = 0.15, *p* = 0.001), Emotional Stability (*r* = 0.16, *p* = 0.001), Social Initiative (*r* = 0.15, *p* = 0.002), and composite score (*r* = 0.19, *p* = 0.000) of MPQ-SF by Pearson correlation. The ANOVA was used to calculate the relationship between categorical demographic variables (gender, size of hometown, educational level, and experience playing musical instruments) and the MPQ-SF dimension scores and composite score. There was statistical significance between size of hometown variable and Cultural Empathy (*r* = 5.58, *p* = 0.004), Open-mindedness (*r* = 13.90, *p* = 0.000), Social Initiative (*r* = 3.53, *p* = 0.030), Flexibility (*r* = 3.09, *p* = 0.047), and composite score (*r* = 9.53, *p* = 0.000). In addition, gender variable was statistical significance with Cultural Empathy (*r* = 4.30, *p* = 0.039), educational level variable was statistical significance with Emotional Stability (*r* = 3.06, *p* = 0.048).

**TABLE 2:** Preservice music teachers’ demographic characteristic correlations with multicultural personality questionnaire-short form.

Subscale	Cultural Empathy	Open-mindedness	Emotional Stability	Social Initiative	Flexibility	Composite
**Variables**						
Years of studying and teaching	0.081	0.153** ^(Sig.001)^	0.156** ^(Sig.001)^	0.152** ^(Sig.002)^	-0.056	0.193** ^(Sig.000)^
Gender	4.303* (Sig.039)	1.585	1.311	1.872	0.401	0.045
Size of hometown	5.581* (Sig. 004)	13.901* (Sig. 000)	2.084	3.526* (Sig. 030)	3.086* (Sig.047)	9.531* (Sig.000)
Educational level	1.451	1.256	3.062* (Sig.048)	1.361	0.666	1.928
Experience playing musical instruments	1.621	2.510	0.804	0.329	0.529	2.478

*Significant at 0.05.

**Significant at 0.01.

The results show in Table 3 that preservice music teachers’ gender variable only statistically significantly differed in the Cultural Empathy, *F*(1,431) = 4.30, *p* = 0.039. The preservice music teachers’ educational level variable was statistically significant on the Emotional Stability, *F*(2,430) = 3.06, *p* = 0.048. It is clear that the preservice music teachers’ hometown size had statistical significance on the Cultural Empathy, *F*(2,430) = 5.58, *p* = 0.004, Open-mindedness, *F*(2,430) = 13.90, *p* = 0.000, Social Initiative, *F*(2,430) = 3.53, *p* = 0.030, Flexibility, *F*(2,430) = 3.09, *p* = 0.047, and MPQ-SF composite scale, *F*(2,430) = 9.53, *p* = 0.000. Therefore, preservice music teachers’ hometown size and years of studying and teaching variables affect their multicultural personality traits.

**TABLE 3:** Results of analysis of variance categorical demographics variables and MPQ-SF.

	Source	SS	d.f.	MS	F	Sig.
**CE**	**Gender**					
	Between Groups	1.552	1	1.552	4.303*	0.039
	Within Groups	155.457	431	0.361		
**Om**	**Size of hometown**					
	Between Groups	3.973	2	1.986	5.581*	0.004
	Within Groups	153.037	430	0.356		
	**Size of hometown**					
	Between Groups	11.640	2	5.820	13.901*	0.000
	Within Groups	180.024	430	0.419		
**ES**	**Educational level**					
	Between Groups	2.358	2	1.179	3.062*	0.048
	Within Groups	165.541	430	0.385		
**SI**	**Size of hometown**					
	Between Groups	1.668	2	0.834	3.526*	0.030
	Within Groups	101.730	430	0.237		
**Fl**	**Size of hometown**					
	Between Groups	2.236	2	1.118	3.086*	0.047
	Within Groups	155.823	430	0.362		
**MPQ-SF**	**Size of hometown**					
	Between Groups	1.641	2	0.821	9.531*	0.000
	Within Groups	37.020	430	0.086		

*Significant at 0.05.

Hierarchical regression analysis was used to explore whether gradually increasing the variables gender, educational level, and experience playing musical instruments could improve the effect of size of hometown and years of studying and teaching on the MPQ-SF. The size of hometown and years of studying and teaching variables statistically significantly predicted MPQ-SF (see Model 1 in Table 4), *R*^2^ = 0.068, *F*(2,430) = 15.685 (*p* < 0.001), explaining 6.8% of the MPQ-SF variance. The Model 2 indicated that experience playing musical instruments variable explained 0.1% in the MPQ-SF. The final model (see Model 4 in Table 4) incorporated five variables: gender, size of hometown, years of studying and teaching, educational level, and experience playing musical instruments, with statistically significant *R*^2^ = 0.069, *F*(5,427) = 6.336 (*p* < 0.001). This means that the demographic variables explain 6.9% of the variance in the MPQ-SF. In contrast, the net contribution to the MPQ-SF variance by gender, educational level, and experience playing musical instruments (0.1%) was much lower than the small benchmark (0.5%) and hence negligible (Wei and Hu, 2019).

**TABLE 4:** Hierarchical regression predicting MPQ-SF:model summary.

	Model 1 Size of hometown & Years of studying and teaching	Model 2 Experience playing musical instruments	Model 3 Educational level	Model 4 Gender
*R* ^2^	0.068	0.069	0.069	0.069
*F*	15.685	10.482	7.853	6.336
Δ*R*^2^	0.068	0.001	0.000	0.000
Δ*F*	15.685	0.320	0.137	0.037

*For Models 2–4, the variable underneath model indicates that it was the newly added predictor in this particular model (or block as called in SPSS).*

Therefore, size of hometown, years of studying and teaching emerged as two essential predictors for MPQ-SF. Table 5 shows the regression coefficients for preservice music teachers’ demographic variables on the MPQ-SF. The results indicate that with other factors in the model held constant, the unstandardized beta = 0.085, which means a one-point increase in the size of hometown score increases the MPQ-SF score by 0.085 points. The unstandardized beta = 0.058 for years of studying and teaching, which means a one-point increase in the size of hometown score increases the MPQ-SF score by 0.058 points. The size of hometown variable indicated a contribution of β = 0.18 with the significance level of 0.00. The years of studying and teaching variable indicated a contribution of β = 0.16 with a significance level of 0.00. The MPQ-SF score was statistically significantly positively correlated with the size of hometown and years of studying and teaching. Non-significant correlations were indicated between the MPQ-SF and gender, educational level, and experience playing musical instruments.

**TABLE 5:** Regression coefficients for demographic characteristic on the multicultural personality questionnaire-short from.

Model	Unstandardized Coefficients		Standardized Coefficients	*t*	Significant
	B	Std. Error	Beta		
(Constant)	3.485	0.099		35.281	0.000
Gender	−0.006	0.031	−0009	−0.193	−0.847
Size of Hometown	0.085	0.023	0.178	3.725	0.000
Educational Level	0.014	0.038	0.018	0.372	0.710
Years of studying and teaching	0.058	0.018	0.159	3.267	0.001
Experience playing musical instruments	−0.009	0.017	−0.026	−0.563	0.574

## Discussion

This study was a quantitative study in which data were analyzed using mean, standard deviation, Pearson product-moment correlation, analysis of variance (ANOVA), and regression analysis to effectively assess the level of the multicultural personality of preservice music teachers and the factors that influence the formation of multicultural personality.

The results of this study indicate that preservice music teachers in Sichuan Province have a Mid-Range level in multicultural personality, based on the MPQ-SF (*M* = 3.36). Preservice teachers’ multicultural personality was highest in the Cultural Empathy dimension and lowest in the Flexibility dimension, which is consistent with previous research results (Polat, 2009; Polat and Metin, 2012; Polat and Barka, 2014; Eskici, 2016; Fietzer et al., 2020). Although the teachers sampled in previous research were trained in different countries, they all scored high on the social-perceptual traits (Cultural Empathy, Open-mindedness, and Social Initiative) and low on stress-buffering traits (Emotional Stability and Flexibility). This finding means preservice teachers view cross-cultural situations as challenges. While cross-cultural situations are opportunities to explore and understand society, teachers need to improve their resilience in facing cross-cultural environment threats. Moreover, preservice music teachers reported that they could reasonably keep peace and focus on problem-solving when facing stress or uncertain situations. However, their emotions may fluctuate in high-stress conditions or due to a lack of social support, and they may even experience fear or tension. When these teachers face trouble during multicultural music education, they may need time or additional help to solve the problem. However, within the context of globalization, teachers need to have a high-level multicultural personality to effectively deal with cultural differences in their work, study, and personal lives. Multicultural education would be beneficial to improve preservice teachers’ multicultural personality traits (Polat and Barka, 2014; Eskici, 2016; Polat, 2018). Therefore, more training and support in multicultural music education is essential for preservice music teachers in Sichuan Province, China.

Preservice music teachers’ hometown size significantly affects their multicultural personality, contrary to the findings of Polat (2009, 2018). Preservice music teachers from large cities are more multicultural than those from rural areas. There are various reasons that the size of hometown influences preservice music teachers’ multicultural personality. One possible explanation is that preservice teachers from larger cities have different opportunities to experience multiculturalism in life than those from rural communities. First, China is a developing country, and the differences between urban and rural areas in administration, economy, education, and medical care form an invisible Great Wall (Knight and Gunatilaka, 2010). The rural economy is backward, and children live in a relatively monocultural environment, making it difficult for them to contact different cultural groups. These early experiences are crucial to the formation of preservice music teachers’ personalities. Secondly, there is a significant educational gap between urban and rural areas in China, even though the Chinese government has promulgated the 9-year compulsory education and the national training plan for rural teachers and vigorously supports rural education resources. The lack of multicultural education in rural areas and the low intercultural ability of students caused by the educational differences between urban and rural areas are problems that cannot be ignored. Lastly, students from rural areas experience more stress than same-age students from metropolitan and urban parts of China (Durkin et al., 2003; Melander, 2018). This excessive pressure affects rural students, leading them to be passive and relatively closed off during social activities. Thus, relatively weak stress-buffering traits (Emotional Stability and Flexibility) were formed in their multicultural personality. The significant difference between urban and rural areas result in statistically significant differences among preservice music teachers in the early stages of their multicultural personality formation. Therefore, the size of the hometown variable statistically significantly affects the preservice music teachers’ multicultural personality, which is the expected result of this study.

The number of years spent studying and teaching also statistically significantly influenced preservice music teachers’ multicultural personalities. Similar to previous studies (Kim, 2017; Hofhuis et al., 2020), the results of this study indicate that learning can improve intercultural competence. Various reasons can explain this impact. First, individuals will acquire a pattern when they are 20–30 years old. What they learned before the age of 20 will become their personal characteristics through their environment and experiences over the next few years (McCrae and Costa, 2003). Therefore, an individual’s multicultural education and experience during the early stages will influence the formation of a multicultural personality. Second, multicultural education effectively improves the preservice teachers’ multicultural personality traits (Polat and Barka, 2014; Eskici, 2016; Polat, 2018). Learning large amounts of multicultural knowledge can help preservice teachers improve their cross-cultural competence. Understanding the cultural differences of different ethnic groups can help preservice teachers better understand their own national culture and enhance their cross-cultural confidence. This insight makes teachers more flexible, open-minded, and inclusive in multicultural social activities and more able to carry out intercultural communication successfully. Third, teaching is a multicultural experience, and individual differences in years of teaching experience form unique personality characteristics (Sharma, 2005). Teaching multicultural knowledge is also a process of self-improvement. More knowledge and experience can impact teachers’ intercultural ability and confidence (Wang, 2004). It is worth noting that the years spent studying and teaching had a more significant impact on the multicultural personality traits of Open-mindedness, Emotional Stability, and Social Initiative. Preservice teachers with rich studying and teaching experience had a more open and unbiased attitude to facing unknown situations. Therefore, years of studying and teaching is a significant variable to preservice music teachers’ multicultural personality formation, which is the expected result of this study.

In this study, preservice music teachers’ gender showed no significance with their multicultural personality but in previous studies, male and female teachers had significantly different Cultural Empathy and Emotional Stability in their multicultural personalities (Budaev, 1999; Eskici, 2016). The main reason may be the cultural differences of the samples. Although gender differences have been shown to be related to many personality traits (Weisberg et al., 2011), studies have shown that in developed Western cultures where gender roles are not so traditional, gender differences in personality tend to be greater (Costa et al., 2001; Schmitt et al., 2008). In addition, ethnicity can moderate gender differences, making White gender differences stronger than that of Asians (Weisberg et al., 2011). Age also has a moderating effect on gender differences (Soto et al., 2011). As age increases, the gender gap between men and women becomes wider (Weisberg et al., 2011). Therefore, perhaps due to cultural differences, religion, age, and other factors, the multicultural personality of the Chinese preservice music teachers participating in the survey did not differ significantly in terms of gender.

Various studies have emphasized that education is essential for improving people’s multicultural personality characteristics (Polat, 2018). However, in this study, the educational level of Chinese preservice music teachers had no significant impact on their multicultural personality. The most reasonable explanation is that multicultural music education has not been fully developed in China (Lasauskiene and Sun, 2019). Due to the lack of resources for multicultural music education, China’s multicultural music education has not entirely penetrated higher education. However, traditional music education does not include world music culture and cannot improve the multicultural personality level. In this study, the Mid-Range level multicultural personality of preservice music teachers also confirms the limitations of China’s multicultural music education. Therefore, expanding and implementing multicultural music education is necessary in Chinese music education (Wu, 2020; Wu and Luo, 2021).

Preservice music teachers’ experience playing musical instruments also has no significant impact on their multicultural personality, and this result is consistent with previous studies (Welborn, 2012; Mihajlovski, 2013; Girgi, 2017). Influenced by the European music education’s monism, Chinese music education has traditionally always centered on Western music (Kuang and Cao, 2001; Guan, 2006; Pan, 2020). Research has shown that teachers who have only learned Western musical instruments are more Eurocentric than teachers who have learned all about multicultural music education (Zhang, 2007). China’s music education has changed from Western music-centric theory to multicultural music education (Fan, 2004; Wu and Luo, 2021).

Overall, in this study, the level of the multicultural personality of preservice music teachers was Mid-Range, and their size of hometown and years of studying and teaching were statistically significant in shaping their multicultural personality. This is strongly related to the cultural background of the Sichuan region and the difference between urban and rural education. This study suggests new ideas and directions for developing multicultural music education and music teacher training in China. Cultivating the multicultural personality of preservice music teachers may effectively promote multicultural music education.

## Conclusion

Research shows that Chinese preservice music teachers have a Mid-Range level of multicultural personality due to the lack of Flexibility and Emotional Stability, which are helpful in addressing cultural differences in the face of unknown multicultural conditions. Preservice music teachers whose hometowns are big cities had a higher multicultural personality level than those in rural areas. The amount of time a teacher has spent studying and teaching had a statistically significant influence on their multicultural personalities. Gender, educational level, and experience playing musical instruments had no statistically significant effect on the multicultural personality of preservice music teachers.

In summary, the multicultural personality level of preservice music teachers in Sichuan Province, China, needs improvement, which means multicultural music education requires comprehensive implementation in China. Although the Chinese government promotes multicultural education, whether it has achieved significant results remains to be investigated. Therefore, teacher training programs must address the insufficiencies of teachers’ multicultural personality traits, conduct comprehensive multicultural education and training, improve teachers’ teaching ability to students of different cultural backgrounds, and solve cross-cultural problems.

## Limitations and Recommendations

Our research focus was preservice music teachers in Sichuan Province, China. However, the results cannot represent all preservice music teachers’ current multicultural personality levels in China. Therefore, in future research, different samples need to be verified. In addition, this research fills the gap in the research on the multicultural personality level and influencing factors of preservice music teachers from the perspective of Chinese multicultural music education. But the factors that affect personality are not limited to this, and include other variables such as religion, age, and parental education background. It is necessary to pay attention to the research of variables that have a moderating effect. Therefore, future research needs to investigate the influence of different variables and moderating variables on multicultural personality.

## Data Availability Statement

The raw data supporting the conclusions of this article will be made available by the authors, without undue reservation.

## Author Contributions

Both authors contributed to the conception and design of the study, manuscript revision, and read and approved the submitted version.

## Conflict of Interest

The authors declare that the research was conducted in the absence of any commercial or financial relationships that could be construed as a potential conflict of interest.

## Publisher’s Note

All claims expressed in this article are solely those of the authors and do not necessarily represent those of their affiliated organizations, or those of the publisher, the editors and the reviewers. Any product that may be evaluated in this article, or claim that may be made by its manufacturer, is not guaranteed or endorsed by the publisher.

## References

[B1] Barnatt J. D’Souza L. A. Gleeson A. M. Viesca K. M. Wery J. (2019). Intercultural competence in preservice teacher candidates. *Int. J. Educ. Ref.* 29 211–235. 10.1177/1056787919896866

[B2] Brislin R. W. (1980). Translation and content analysis of oral and written material. *Handb. Cross Cult. Psychol.* 2 349–444.

[B3] Brummett B. R. Wade J. C. Ponterotto J. G. Thombs B. Lewis C. (2007). Psychosocial well-being and a multicultural personality disposition. *J. Counsel. Dev.* 85 73–81. 10.1002/j.1556-6678.2007.tb00446.x

[B4] Budaev S. V. (1999). Sex Differences in the Big Five personality factors: testing an evolutionary hypothesis. *Personal. Individ. Differ.* 26 801–813. 10.1016/S0191-8869(98)00179-2

[B5] Costa P. T. Jr. Terracciano A. McCrae R. R. (2001). Gender differences in personality traits across cultures: robust and surprising findings. *J. Personal. Soc. Psychol.* 81 322–331. 10.1037/0022-3514.81.2.322 11519935

[B6] Durkin S. R. Bascomb A. Turnbull D. Marley J. (2003). Rural origin medical students: how do they cope with the medical school environment? *Aust. J. Rural Health* 11 89–95. 10.1046/j.1440-1584.2003.00512.x 12780499

[B7] Eilam B. Vidergor H. E. (2011). Gifted Israeli students’ perceptions of teachers’ desired characteristics: a case of cultural orientation. *Roeper Rev.* 33 86–96. 10.1080/02783193.2011.554156

[B8] Eryilmaz A. (2014). Perceived personality traits and types of teachers and their relationship to the subjective well-being and academic achievements of adolescents. *Educ. Sci.* 14 2049–2062.

[B9] Eskici M. (2016). Prospective teachers’ personal characteristics to multicultural education. *Universal J. Educ. Res.* 4 102–111. 10.1111/josh.12589 29333643

[B10] Fan Z. Y. (2004). Chinese minority music and its role and status in the world’s multicultural music education. *Chin. Music* 11–14.

[B11] Fietzer A. W. Black N. Ponterotto J. G. Magaldi D. Lipari K. Pratt A. (2020). The Multicultural Personality Inventory–Short Form: development and validation. *Measure. Eval. Counsel. Dev.* 53 165–181. 10.1080/07481756.2019.1691460

[B12] Gawali G. Khattar T. (2016). The influence of multicultural personality on attitude towards religious diversity among youth. *J. Indian Acad. Appl. Psychol.* 42 114–123. 10.1016/j.socscimed.2018.03.011 29626718

[B13] Girgi D. (2017). The relationship between preservice music teachers’ self-efficacy belief in musical instrument performance and personality traits. *Eur. J. Educ. Res.* 16 107–123. 10.14689/ejer.2017.67.7

[B14] Guan J. H. (2005). International Society for Music Education and multicultural music education. *J. Xinjiang Normal University* 26 233–238.

[B15] Guan J. H. (2006). *Comparing Chinese and Western Music and Musical Culture.* China: The Western Shaanxi Normal University Press.

[B16] Gunara S. Sutanto T. S. (2021). Enhancing the intercultural competence development of prospective music teacher education: a case study in Indonesia. *Int. J. High. Educ.* 10 150–157. 10.5430/ijhe.v10n3p150

[B17] Hofhuis J. Jongerling J. Van der Zee K. I. Jansz J. (2020). Validation of the Multicultural Personality Questionnaire Short Form (MPQ-SF) for use in the context of international education. *PLoS One* 15:e0244425. 10.1371/journal.pone.0244425 33370395 PMC7769263

[B18] Jičínská B. A. M. (2014). *Souvislost Citové Vazby a Interkulturní Efektivity [Online]*. Bakalářská práce. Brno: Masarykova Univerzita, Fakulta Sociálních Studií.

[B19] Kaplan L. (1961). *The Relationship between Certain Personality Characteristics and Achievement in Instrumental Music.* New York, NY: New York University.

[B20] Khalilzadeh S. Khodi A. (2018). Teachers’ personality traits and students’ motivation: a structural equation modeling analysis. *Curr. Psychol.* 40, 1635-1650. 10.1007/s12144-018-0064-8

[B21] Kim Y. Y. (2017). “Cross-cultural adaptation,” in *Oxford Research Encyclopedia of Communication*, ed. Nussbaum J. (New York, NY: Oxford University Press), 10.1093/acrefore/9780190228613.013.21

[B22] Knight J. Gunatilaka R. (2010). The rural-urban divide in China: income but not happiness? *J. Dev. Stud.* 46 506–534. 10.1080/00220380903012763

[B23] Korol L. Fietzer A. W. Ponterotto J. G. (2018). The relationship between multicultural personality, intergroup contact, and positive outgroup attitudes toward Asian Americans. *Asian Am. J. Psychol.* 9 200–210. 10.1037/aap0000107

[B24] Kuang H. Cao F. (2001). The important contribution of the basic viewpoints of cultural anthropology to the development of modern European music education and culture. *Chin. Music* 48–51.

[B25] Lasauskiene J. (2018). An innovative pedagogical design of intercultural competence development in music teacher education. *Rural Environ. Educ. Personal* 11 208–214. 10.22616/REEP.2018.025

[B26] Lasauskiene J. Sun Y. (2019). Challenges and visions in school music education: focusing on Chinese and Lithuanian realities. *New Trends Issues Proc. Human. Soc. Sci.* 6 38–46. 10.18844/prosoc.v6i1.4153

[B27] Leibold J. Chen Y. B. (2014). *Minority Education in China: Balancing Unity and Diversity in an era of Critical Pluralism.* Hong Kong: Hong Kong University Press.

[B28] Liang Y. Schartner A. (2020). Culturally mixed group work and the development of students’ intercultural competence. *J. Stud. Int. Educ.* 26 44–60. 10.1177/1028315320963507

[B29] Lukman M. Istiyono E. Kartowagiran B. Retnawati H. Adi H. C. Kistoro H. P. (2021). Effective teachers’ personality in strengthening character education. *Int. J. Eval. Res. Educ. ISSN* 10 512–521. 10.11591/ijere.v10i2.21629

[B30] Ma D. (2006). The concept of implementing the world multicultural music education in the music education major of normal university. *People’s Music* 82–85.

[B31] Ma H. (2013). Research on the construction of diversified music education in universities. *Northern Music* 26–27.

[B32] McCarthy S. K. (2009). *Communist Multiculturalism: Ethnic Revival in Southwest China.* Seattle: University of Washington Press.

[B33] McCrae R. R. Costa P. T. (2003). *Personality in Adulthood: A Five-Factor Theory Perspective.* New York, NY: Guilford Press.

[B34] Melander J. (2018). *Hometown Size and its Relationship to Emotional and Social Adjustment to College.* [Ph.D.thesis]. Murfreesboro, TN: Middle Tennessee State University.

[B35] Mihajlovski Z. (2013). Personality, intelligence and musical instrument. *Croatian J. Educ.* 15 155–172.

[B36] Mikheeva T. B. (2018). “Intercultural competence of multicultural personality of a student,” in *The European Proceedings of Social & Behavioural Sciences (EpSBS)*, ed. Denisova I. V. (London: Future Academy), 109–114.

[B37] Olejárová D. Delalande H. Èervenková M. (2020). Measuring multicultural effectiveness of Slovak and Czech students with the multicultural personality questionnaire. *Czech. Psychol.*

[B38] Pan C. (2020). The distinction between “Multiculturalism” and “Returning to the origin and creating the new” – research on Guan Jianhua’s music education philosophy. *Chin. Music* 2.

[B39] Peng M. G. (2013). *Research on Harmonious Personality Construction of College Students from the Perspective of Modernization*. Doctoral dissertation. Nanjing: Nanjing University of Science & Technology.

[B40] Plugina M. I. Rodionova I. V. Belasheva I. V. Oganyan K. M. Enin V. V. (2021). “Multicultural competence as a factor in updating the cognitive potential of a teacher at a higher school,” in *Modern Global Economic System: Evolutional Development vs. Revolutionary Leap*, eds Popkova E. G. Sergi B. S. (Cham: Springer International Publishing), 527–535.

[B41] Polat S. (2009). Probationary Teachers’ Level of Inclination to Multi-Cultural Education/ Öǧretmen adaylarının çokkültürlü eǧitime yönelik kişilik özellikleri. *Int. Online J. Educ. Sci.* 1 154–164.

[B42] Polat S. (2018). An analysis of the multicultural characteristics of the preservice teachers in terms of the values they have. *Asian J. Educ. Train.* 4 336–346. 10.20944/preprints201807.0099.v1 32283112

[B43] Polat S. Barka T. O. (2014). Preservice teachers’ intercultural competence: a comparative study of teachers in Switzerland and Turkey. *Eur. J. Educ. Res.* 54 19–38. 10.14689/ejer.2014.54.2

[B44] Polat S. Metin M. A. (2012). The relationship between the teachers’ intercultural competence levels and the strategy of solving conflicts. *Procedia Soc. Behav. Sci.* 46 1961–1968. 10.1016/j.sbspro.2012.05.411

[B45] Ponterotto J. G. (2010). Multicultural personality: an evolving theory of optimal functioning in culturally heterogeneous societies. *Couns. Psychol.* 38 714–758.

[B46] Ponterotto J. G. Ruckdeschel D. E. Joseph A. C. Tennenbaum E. A. Bruno A. (2011). Multicultural personality dispositions and trait emotional intelligence: an exploratory study. *J. Soc. Psychol.* 151 556–523. 10.1080/00224545.2010.503718 22017073

[B47] Qian L. Colak F. Z. Agirdag O. (2020). Characteristics, issues, and future directions in Chinese multicultural education: a review of selected research 2000–2018. *Asia Pacific Educ. Rev*. 21 279–294. 10.1007/s12564-020-09624-2

[B48] Rosiers A. Eyckmans J. Heirman W. Carlson T. Van Craeynest K. (2014). Multicultural effectiveness in advanced language learners. *Sci. Total Environ.* 499 99–106.25173866

[B49] Sample D. Hotchkiss S. M. (1971). An investigation of relationships between personality characteristics and success in instrumental study. *J. Res. Music Educ.* 19 307–313.

[B50] Sarraj H. Carter S. Burley H. (2015). Literature review of multicultural instrumentation. *Multicul. Perspect.* 17 225–233. 10.1080/15210960.2015.1088307

[B51] Schmitt D. P. Realo A. Voracek M. Allik J. (2008). Why can’t a man be more like a woman? Sex differences in Big Five personality traits across 55 cultures. *J. Personal. Soc. Psychol.* 94 168–182. 10.1037/0022-3514.94.1.168 18179326

[B52] Shakeel S. Khan M. M. Khan R. A. A. Mujtaba B. G. (2021). Linking personality traits, self-efficacy and burnout of teachers in public schools: does school climate play a moderating role? *Public Organ. Rev.* 22 1–21. 10.1007/s11115-021-00514-8

[B53] Sharma S. (2005). Multicultural education: teachers’ perceptions and preparation. *J. College Teach. Learn.* 2 53–63. 10.19030/tlc.v2i5.1825

[B54] Soto C. J. John O. P. Gosling S. D. Potter J. (2011). Age differences in personality traits from 10 to 65: Big Five domains and facets in a large cross-sectional sample. *J. Pers. Soc. Psychol.* 100 330–348. 10.1037/a0021717 21171787

[B55] The Department of Sports, Health and Art Education, and Ministry of Education (2003). *Primary school music teaching method.*

[B56] The Ministry of Education of the People’s Republic of China [MOE] (2001). *Full-Time Compulsory Education Music Curriculum Standards (Experimental Draft), Homepage.* Beijing: The Ministry of Education of the People’s Republic of China.

[B57] The Ministry of Education of the People’s Republic of China [MoE] (2007). *National Musicology of Higher Education (Teacher Education) Undergraduate Professional Curriculum Guidance Program, Homepage.* Beijing: The Ministry of Education of the People’s Republic of China.

[B58] The Ministry of Education of the People’s Republic of China [MOE] (2011). *Compulsory Education Music Curriculum Standards*, 2011 Edn. Beijing: Beijing Normal University Press.

[B59] Tortop H. S. (2014). Öǧretmen adaylarının üstün yetenekli ve çok kültürlü eǧitime ilişkin tutumları. *Üstün Yetenekliler Eǧitimi ve Araştırmaları Dergisi (UYAD)* 2 16–26.

[B60] Tracy-Ventura N. Dewaele J. M. Köylü Z. McManus K. (2016). Personality changes after the ‘year abroad’?: a mixed-methods study. *Study Abroad Res. Sec. Lang. Acq. Int. Educ.* 1 107–127. 10.1075/sar.1.1.05tra 33486653

[B61] Vaag J. Sund E. R. Bjerkeset O. (2018). Five-factor personality profiles among Norwegian musicians compared to the general workforce. *Musicae Sci.* 22 434–445. 10.1177/1029864917709519

[B62] van der Zee K. van Oudenhoven J. P. (2000). The Multicultural Personality Questionnaire: a multidimensional instrument of multicultural effectiveness. *Eur. J. Personal.* 14 291–309. 10.1002/1099-0984(200007/08)14:4<291::AID-PER377<3.0.CO;2-6

[B63] van der Zee K. van Oudenhoven J. P. (2001). The Multicultural Personality Questionnaire: reliability and validity of self-and other ratings of multicultural effectiveness. *J. Res. Personal.* 35 278–288. 10.1006/jrpe.2001.2320

[B64] van der Zee K. van Oudenhoven J. P. Ponterotto J. P. Fietzer A. W. (2013). Multicultural Personality Questionnaire: development of a short form. *J. Personal. Assess.* 95 118–124. 10.1080/00223891.2012.718302 22966866

[B65] Wang J. (2004). Multicultural education: western minority education and its enlightenment. *Guangxi Ethnic Stud.* 112–117.

[B66] Wang Y. H. (2003). On the training objectives and curriculum structure system of music education major in normal universities. *Chin. Music Educ.* 21–23. 10.1016/j.jvoice.2014.08.013 25704468

[B67] Wei R. Hu Y. (2019). Exploring the relationship between multilingualism and tolerance of ambiguity: a survey study from an EFL context. *Bilingualism* 22 1209–1219. 10.1017/S1366728918000998

[B68] Weisberg Y. J. DeYoung C. G. Hirsh J. B. (2011). Gender differences in personality across the ten aspects of the Big Five. *Front. Psychol.* 2:178. 10.3389/fpsyg.2011.00178 21866227 PMC3149680

[B69] Welborn D. C. (2012). *Adult Community Bands and Personality Type as Defined by the Myers-Briggs: A Study of the Personality Types and Music Participation Preferences of Adult Musicians.* Hattiesburg, MS: The University of Southern Mississippi.

[B70] Win S. Yee H. O. (2018). An investigation into the personality, attitude, and teacher effectiveness of in-service teachers. *Univ. Res. J.* 11 289–306.

[B71] Wong K. Y. Pan K. C. Shah S. M. (2016). General music teachers’ attitudes and practices regarding multicultural music education in Malaysia. *Music Educ. Res.* 18 208–223. 10.1080/14613808.2015.1052383

[B72] Wu W. Y. Bodigerel-Koehler M. (2013). The mediating effects of cross-cultural dynamic competencies on the relationship between multicultural personality and cross-cultural adjustment. *Int. J. Hum. Res. Manage.* 24 4026–4045. 10.1080/09585192.2013.781518

[B73] Wu Y. (2020). The reality and reflection of Multicultural music education in China. *Music Res.* 9 111–117.

[B74] Wu Y. Luo Q. (2021). On the Function of music education in the concept of multi-culture of music anthropology and its Practice in China. *Res. Music Cult.* 63–73.

[B75] Ye Q. (2020). *The Effect of Multicultural Personality and Contact on Chinese Domestic Students’ Attitudes Toward International Students In China.* [Ph.D.thesis]. China: Shanghai International Studies University.

[B76] Zhang Y. (2007). *Attitude and Teacher training of Music Teachers in multicultural Background.* [Ph.D.thesis]. Lanzhou, China: Northwest Normal University.

